# Axial spondyloarthritis and breastfeeding: a prospective study

**DOI:** 10.1186/s13006-025-00714-8

**Published:** 2025-03-29

**Authors:** Emma Hortemo Grøntvedt, Ingrid Mørdre, Marianne Wallenius, Hege Svean Koksvik, Carina Gøtestam Skorpen

**Affiliations:** 1 Faculty of Medicine and Health Sciences, Norwegian University of Science and Technology (NTNU), Trondheim, Norway; 2 Institute of Neuromedicine and Movement Science, Norwegian University of Science and Technology (NTNU), Trondheim, Norway; 3 Department of Rheumatology, Norwegian National Network for Pregnancy and Rheumatic Diseases (NKSR), St Olavs Hospital, Trondheim University Hospital, Trondheim, Norway; 4 Department of Health Sciences, Norwegian University of Science and Technology (NTNU), Ålesund, Norway; 5 Department of Rheumatology, Ålesund Hospital, Ålesund, Norway

**Keywords:** Breastfeeding, Axial Spondyloarthritis, Quality of life, Womens health, Register study, Postpartum period, Inflammatory rheumatic diseases, Prospective study

## Abstract

**Background:**

There is sparse literature on the topic of breastfeeding in women with axial spondyloarthritis (axSpA). Our aim was to obtain more knowledge about variables affecting breastfeeding for women with axSpA.

**Methods:**

This prospective study used data from the nationwide quality register RevNatus, which collects pregnancy-related data in women with inflammatory rheumatic diseases from Norwegian outpatient clinics in rheumatology. Data were collected during January 2016 to August 2023, reporting on 436 pregnancies in 363 patients with axSpA. The study eventually included 417 births in 350 women. Breastfeeding and non-breastfeeding women were compared at six weeks, six and twelve months postpartum*.* We compared the groups regarding demographic and obstetric data, neonatal outcome, disease characteristics, medical treatment and self-reported data on pain, fatigue and disease burden. Information on breastfeeding was registered at least once during the follow-up postpartum. Disease activity was measured by Ankylosing Spondyloarthritis Disease Activity Index-CRP (ASDAS-CRP) and Bath Ankylosing Disease Activity Index (BASDAI).

**Results:**

The proportion of patients breastfeeding at the postpartum follow ups was 86% (347 women) at six weeks, 70% (221 women) at six months, and 38% (104 women) at twelve months, respectively.

A larger proportion of the non-breastfeeding group had delivered with caesarean section (C-section), 24 of 59 (41%) non-breastfeeding vs 70 of 347 (20%) breastfeeding women. ASDAS-CRP was higher for the non-breastfeeding group at six weeks (2.6 vs 2.2) and at six months (2.6 vs 2.1), and BASDAI was higher for the same group at six months (4.1 vs 3.2). CRP (mg/L) was significantly higher among the non-breastfeeding at six months (5.3 vs 3.3). VAS pain scores were higher for the non-breastfeeding group at six weeks (41.0 vs 31.6) and six months (43.9 vs 31.0). VAS fatigue was higher for the same group at six months (46.8 vs 37.8).

**Conclusion:**

Our results suggest that particular attention should be given regarding breastfeeding to women with active inflammatory disease and those who have undergone C-section.

## Background

Axial spondyloarthritis (axSpA) is a group of inflammatory rheumatic diseases characterized by inflammation of the spine [1]. Other common symptoms include enthesitis, arthritis and dactylitis [2]. The onset is around 20–30 years of age. While non-radiographic axSpA (nr-axSpA) affects both sexes equally, radiographic axSpA (r-axSpA) has a male/female ratio of 2:1 [3].

AxSpA is a chronic disease that lasts a lifetime. The course of the disease is characterized by fluctuations in disease activity, and the patient will experience periods of improvement and deterioration of the disease [4].

The treatment aims to prevent progression of the disease, maintain control of symptoms and inflammation, and improve health related quality of life [3]. Exercise and physiotherapy are cornerstones in the treatment of axSpA. Non-Steroidal Anti-Inflammatory Drugs (NSAIDs) reduce symptoms like pain and stiffness of the back and joints. In more severe cases, disease modifying anti rheumatic drugs (DMARDs) including Tumor Necrosis Factor-inhibitors (TNFi) may be used.

Sulfasalazin, a conventional synthetic DMARD (csDMARD), is compatible with pregnancy and breastfeeding [5–7]. TNFi, which are biologic DMARDs (bDMARDs), are often the recommended treatment for women with axSpA during pregnancy and breastfeeding [5–7]. This includes adalimumab, certolizumab, etanercept, golimumab and infliximab. The IL-17 inhibitor secukinumab, may be used during pregnancy and lactation [6]. Methotrexate (MTX), a conventional csDMARD, is not compatible with pregnancy, and has traditionally been considered not compatible with lactation [5–7].

Data from the Norwegian nationwide quality register RevNatus [8] has shown that disease activity during pregnancy and one year postpartum in women with axSpA was stable, although the disease activity and self-reported pain was highest in the second trimester [1]. Previous publications regarding axSpA and breastfeeding have dealt with medication during and after pregnancy, and how this may have affected both the mother and the child [9, 10]. A previous study has shown that fewer women with spondyloarthritis (SpA) breastfeed compared to women without the disease [11]. To our knowledge, no previous studies have examined if disease activity or self-reported health status have an impact on breastfeeding in women with axSpA.

The aim of this study was to compare the proportion of women with axSpA who breastfed and those who did not, at respectively six weeks, six and twelve months postpartum. We compared the breastfeeding and non-breastfeeding women with regards to demographic and disease characteristics, medical treatment, pregnancy outcomes and self-reported pain, fatigue and disease burden.

## Methods

### The RevNatus-register

This prospective study used data collected in RevNatus, a consent-based nationwide quality register [8]. The register collects pregnancy-related information in women with inflammatory rheumatic diseases, including axSpA, from twenty outpatient clinics in rheumatology in the Norwegian public healthcare system. This information includes demographic data, disease activity, self-reported health status, use of medication and selected pregnancy outcomes. Gestational age at delivery, infant birth weight, delivery method and information about specified pregnancy complications are registered during the visit 6 weeks postpartum. Patients are asked to be included during ordinary visits at the rheumatology outpatient clinic. All participants are above the age of 16, diagnosed with an inflammatory rheumatic disease and are planning pregnancy or are already pregnant. Data are collected on seven different occasions: before pregnancy, in every trimester during pregnancy, and at six weeks, six months and twelve months postpartum.

### Patient population

Data were collected in the period 1 January 2016 to 31 August 2023. As of 31 August 2023, 436 pregnancies occurred in 363 axSpA patients, with sixtytwo women registered with more than one birth. We utilized data registered at time of inclusion in RevNatus, and at six weeks, six and twelve months postpartum. All the patients in our population were diagnosed with axSpA by a rheumatologist, and the majority met the classification criteria. As some women were registered with more than one birth, each birth was considered as an independent case, breastfeeding or not breastfeeding. We excluded births with no data registered regarding breastfeeding. Births with breastfeeding data from one or more postpartum follow-ups were included.

**Fig. 1:**
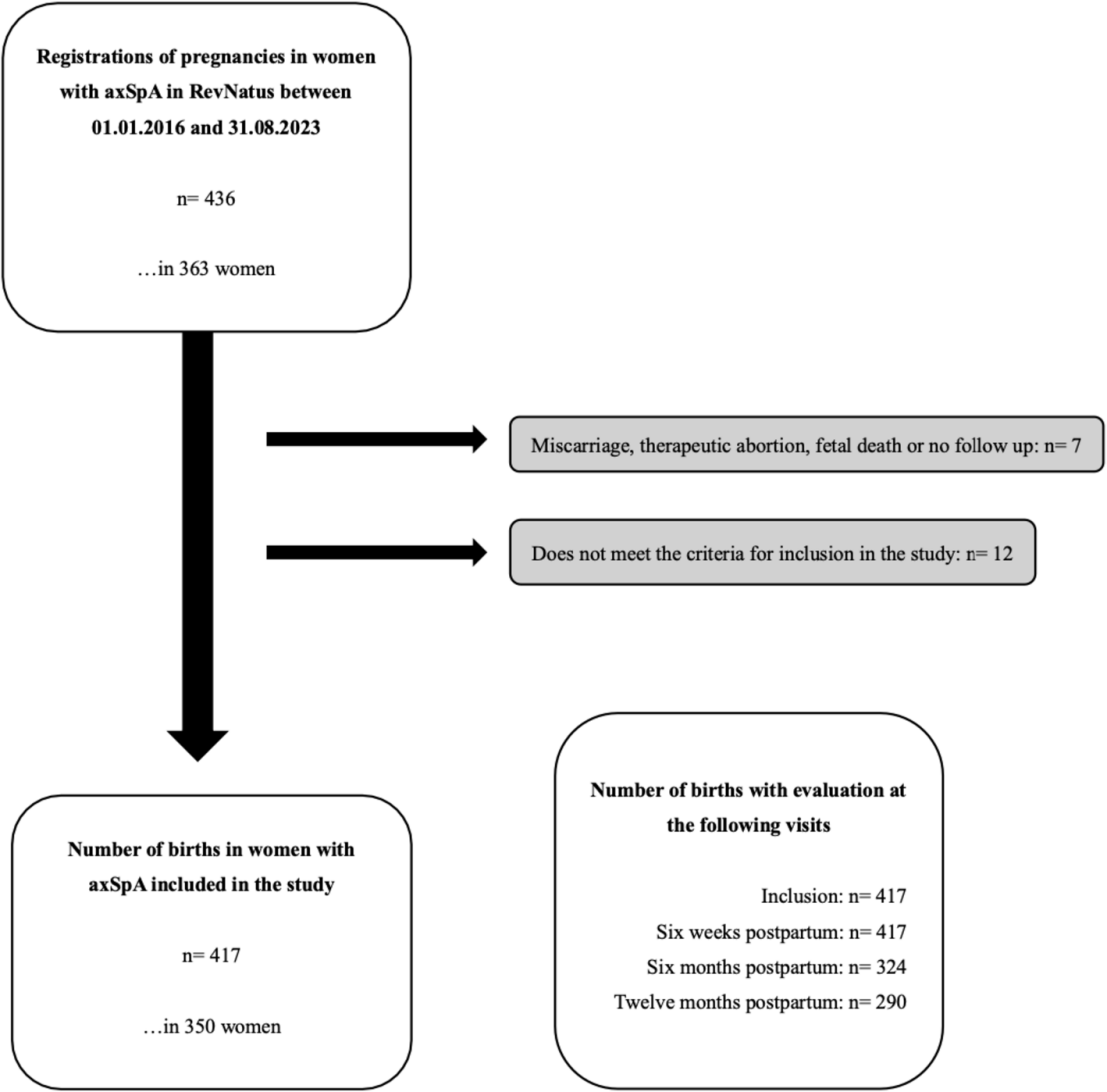
Flowchart of inclusion and exclusion of patients and births in the study. A total of 417 births were eventually included in the study. Twelve births had no data registered regarding breastfeeding at any point in time and did not meet the criteria for inclusion. Seven pregnancies were excluded due to miscarriage, therapeutic abortion, fetal death or no follow up registered for the birth. Reasons behind loss to follow up are unknown

As a result, a total of 417 births in 350 women were included in the study. See Fig. 1. All women attended the six weeks postpartum follow up for all births, whereas the six and twelve month follow ups had a lower attendance, see Table 1.

**Table 1:** Number and proportion of breastfeeding at follow ups postpartum, reported as n (%)

	**n**	**Missing**	**Breastfeeding**	**Not breastfeeding**
6 weeks	417	11	347 (85.5)	59 (14.5)
6 months	324	8	221 (69.9)	95 (30.1)
12 months	290	17	104 (38.1)	169 (61.9)

### Data collection and outcome variables

Demographic data included maternal age, Body Mass Index (BMI), use of tobacco, level of education and work status, exercise, living situation and previous live births (yes/no). To evaluate disease activity, we used validated disease activity assessments Bath Ankylosing Disease Activity Index (BASDAI) [12], Ankylosing Spondyloarthritis Disease Activity Index (ASDAS) [12] in addition to C-reactive protein (CRP). We also included self-reported data on the visual analogue scales (VAS) pain, VAS fatigue and VAS total disease burden (VAS total). Obstetric and neonatal outcomes were registered at the visit six weeks postpartum. This included date of birth, gestational age, birth weight, delivery method (vaginal delivery or caesarean section) and complications during pregnancy, including preeclampsia, eclampsia and Hemolysis, Elevated Liver enzymes and Low Platelets (HELLP) syndrome. All of these hypertensive pregnancy complications were combined to form one single variable. Self-reported questions regarding breastfeeding were registered at respectively six weeks, six and twelve months postpartum (yes/no). Data concerning breastfeeding did not specify whether the woman breastfed exclusively or partially. We also included information about the use of conventional synthetic DMARDs (sulfasalazine and methotrexate) and biologic DMARDs (TNF-inhibitors and the IL-17 inhibitor secukinumab) at inclusion and after birth.

BASDAI is a measure of self-reported disease activity, a questionnaire consisting of six items. These items include back pain, fatigue, peripheral joint pain and swelling, localized tenderness, and duration and severity of morning stiffness [12]. Each question is scored by the patient on a scale from 0–10 (numeric scale or VAS). A total score out of 10 is calculated (0 = no disease activity, 10 = maximum disease activity), with a score of 4 being used as a cut off for active disease [12].

ASDAS-CRP is an index which, in addition to self-reported items, includes an objective measure of inflammation. Five variables are combined to give a single disease score: back pain, duration of morning stiffness, peripheral joint pain and/or swelling and general well-being, as well as the inflammation marker CRP [12]. Self-reported items are scored on a scale from 0–10 (numeric scale or VAS) and are combined with the objective measure to give a total disease activity score on a continuous scale (from 0 with no defined upper end) [12]. Four disease activity states have been defined from this, where a score of < 1.3 separates inactive and moderate disease activity, > 2.1 is considered high disease activity and > 3.5 very high disease activity [13]. We used the cut-off ≥ 1.3 to define active disease according to ASDAS-CRP. Cut-offs for clinical improvement have also been defined, with a change in score of ≥ 1.1 being a clinically important improvement [13].

In addition to BASDAI and ASDAS-CRP, we also utilized CRP alone, as it is a simple and objective measure of active inflammation. Normal levels of CRP are defined as less than 5 mg per liter.

The patients reported subjective pain, fatigue and total disease burden on a VAS scale from 0 to 100, with 0 being no pain/fatigue/disease burden and 100 being extreme pain/fatigue/disease burden.

### Data and statistical analysis

The statistical analyses were conducted using IBM SPSS Statistics version 29. We defined the statistical significance level as two-sided p-value ≤ 0.05, with no adjustments made for multiple comparison. The values for each variable were described as mean ± SD. We used independent student T-tests to compare the breastfeeding and non-breastfeeding women for the continuous variables that were normally distributed. Mann–Whitney-U tests were used to estimate the p-value of the continuous variables that were not normally distributed. We used Chi-square tests or Fisher exact tests to compare frequencies of the categorical variables, depending on the sample sizes.

## Results

### Patient inclusion data

A total of 436 pregnancies in 363 women were recruited and retained for our study. Out of these, 417 births in 350 women were included in our final cohort. Table 1 shows the number and proportion of breastfeeding at six weeks, six and twelve months postpartum. Background characteristics such as demographic data, disease duration, characteristics of the disease and use of medication are shown in Table 2. The number of births with information on each variable is provided in Table 2.

**Table 2:** Patient background and disease-related characteristics collected at time of inclusion

**Number of included births in the study**	n	
Age, mean (SD)	417	30.7 (4.5)
Age ≥ 35 years (%)		82 (19.7)
Missing	0	
Previous live birth (%)	390	206 (52.8)
Missing	27	
Pregnant (%)	417	270 (64.7)
Missing	0	
BMI kg/m^2^ mean (SD)	401	25.7 (4.8)
Underweight (< 18.5) (%)		4 (1.0)
Normal weight (18.5–24.9) (%)		206 (51.4)
Overweight (≥ 25.0) (%)		191 (47.6)
Obesity (≥ 30.0) (%)		73 (18.2)
Missing	16	
Tobacco ^a^ (%)	375	33 (8.8)
Missing	42	
Educational level ^b^ (%)	407	
Low		8 (2.0)
Intermediate		114 (28.0)
High		285 (70.0)
Missing	10	
Working fulltime or part-time (%)	408	309 (75.7)
Missing	9	
Exercising regularly^c^(%)	306	218 (71.2)
Missing	111	
Living situation	403	
Living with someone (%)		390 (96.8)
Living alone (%)		13 (3.2)
Missing	14	
**Disease related characteristics**		
Disease duration, years, mean (SD)	380	4.5 (3.9)
Missing	37	
Classification criteria met (%)	417	389 (93.3)
Missing	0	
HLA-B27 positive (%)	340	271 (79.7)
Missing	77	
CRP mg/L mean (SD)	382	5.8 (7.1)
CRP ≥ 5 (%)		140 (36.6)
Missing	35	
BASDAI, mean (SD)	348	3.4 (2.3)
BASDAI ≥ 4 (%)		132 (37.9)
Missing	69	
ASDAS-CRP, mean (SD)	253	2.2 (0.9)
ASDAS-CRP ≥ 1.3 (%)		206 (81.4)
Missing	164	
VAS scores (0–100 mm) mean (SD)		
Pain	341	32.4 (25.1)
Missing	76	
Fatigue	328	50.0 (30.7)
Missing	89	
Total	339	35.8 (25.7)
Missing	78	
**Medications**		
csDMARDs (%)	417	27 (6.5)
Sulfasalazine		22 (5.3)
Methotrexate		5 (1.2)
Missing	0	
TNF-inhibitors (%)	417	116 (27.8)
Missing	0	
Secukinumab (%)	417	0 (0.0)
Missing	0	

*n* number of available data on variable, *BMI* body mass index, *HLA* human leukocyte antigen, *CRP* C-reactive protein, *VAS* visual analogue scale, *BASDAI* bath ankolysing spondylitis disease activity index, *ASDAS* ankylosing spondylitis disease activity score, *csDMARDs* conventional synthetic disease-modifying anti-rheumatic drugs, *TNF-inhibitors* tumor necrosis factor inhibitors

^a^smoking and snuff use

^b^education level: low = elementary school, intermediate = high school/vocational education, high = college/university

^c^at least once a month

### Six weeks postpartum (Table 3)

Three hundred forty seven women (86%) were breastfeeding at the six weeks postpartum visit. A higher proportion of non-breastfeeding women had delivered with C-section, compared to breastfeeding women (41% vs 20%). The breastfeeding women had a higher mean age than the non-breastfeeding women (31.9 vs 30.5). There were more women breastfeeding among those with high educational level (74.3% vs 46.6%).

We also observed that the non-breastfeeding group had higher disease activity scores, with a mean ASDAS-CRP-score of 2.6 compared to 2.2 in the breastfeeding group. A larger percentage of the non-breastfeeding women had active disease according to their ASDAS-CRP-score (94% vs 80%). We observed the same regarding self-reported health status for VAS pain-score and VAS total-score.

**Table 3:** Differences in breastfeeding and non-breastfeeding in mothers with axSpA six weeks after birth

	**n**	Breastfeeding *N*= 347	**n**	**Non-breastfeeding** *=N* = 59	*p*-value	**test value**†	**df**
Age, mean (SD)	347	31.9 (4.3)	59	30.5 (4.7)	0.03	2.22	404
Age ≥ 35 years (%)		93 (26.8)		10 (16.9)	0.1	2.59	1
Missing	0						
Previous live birth (%)	323	167 (51.7)	56	31 (55.4)	0.6	0.26	1
Missing	27						
BMI kg/m^2^ mean (SD)	318	26.6 (4.5)	50	27.8 (5.4)	0.08	−1.77	366
Underweight (< 18.5) (%)		1 (0.3)		0 (0.0)	1.0		
Normal weight (18.5–24.9) (%)		128 (40.3)		18 (36.0)	0.6	0.33	1
Overweight (≥ 25.0) (%)		189 (59.4)		32 (64.0)	0.5	0.38	1
Obesity (≥ 30.0) (%)		64 (20.1)		15 (30.0)	0.1	2.50	1
Missing	38						
Tobacco ^a^ (%)	315	11 (3.5)	53	5 (9.4)	0.06		
Missing	38						
Educational level ^b^	339		58				
Low (%)		5 (1.5)		2 (3.4)	0.27		
Intermediate (%)		82 (24.2)		29 (50.0)	< 0.001	16.38	1
High (%)		252 (74.3)		27 (46.6)	< 0.001	18.30	1
Missing	9						
Working fulltime or part-time (%)	345	28 (8.1)	59	3 (5.1)	0.6		
Missing	2						
Exercising regularly ^c^ (%)	259	97 (37.5)	43	22 (51.2)	0.09	2.90	1
Missing	104						
CRP mg/L mean (SD)	290	5.5 (8.0)	45	6.0 (9.5)	0.7	−0.43	333
Missing	71						
BASDAI mean (SD)	277	3.0 (2.3)	45	3.6 (2.4)	0.1	−1.62	320
BASDAI ≥ 4 (%)		85 (30.7)		19 (42.2)	0.1	2.36	1
Missing	84						
ASDAS-CRP mean (SD)	201	2.2 (1.0)	36	2.6 (1.0)	0.03	−2.19	235
ASDAS-CRP ≥ 1.3 (%)		161 (80.1)		34 (94.4)	0.04	4.31	1
Missing	169						
VAS scores (0–100 mm) mean (SD)							
Pain	291	31.6 (26.4)	50	41.0 (29.7)	0.03	−2.18	
Missing	65						
Fatigue	281	36.9 (29.4)	48	43.5 (29.2)	0.1	−1.50	
Missing	77						
Total	292	35.8 (27.7)	49	46.5 (27.8)	0.01	−2.54	
Missing	65						
Prematurity (%)	347	17 (4.9)	59	7 (11.9)	0.07		
Missing	0						
Low birthweight ^d^ (%)	344	14 (4.1)	57	3 (5.3)	0.7		
Missing	5						
C-section (%)	347	70 (20.2)	59	24 (40.7)	< 0.001	11.92	1
Missing	0						
Preeclampsia ^e^ (%)	338	21 (6.2)	57	4 (7.0)	0.8		
Missing	11						
csDMARDs (%)	347	22 (6.3)	59	4 (6.8)	0.8		
Sulfasalazine		22 (6.3)		4 (6.8)	0.8		
Methotrexate		0 (0.0)		1 (1.7)	0.1		
Missing	0						
TNF-inhibitors (%)	347	106 (30.5)	59	17 (28.8)	0.8	0.72	1
Missing	0						
Secukinumab (%)	347	4 (1.2)	59	0 (0.0)	1.0		
Missing	0						

*n* number of available data on variable, *df* degrees of freedom, *BMI* body mass index, *CRP* C-reactive protein, *BASDAI* bath ankolysing spondylitis disease activity index, *ASDAS* ankylosing spondylitis disease activity score, *VAS* visual analogue scale, *csDMARDs* conventional synthetic disease-modifying anti-rheumatic drugs, *TNF-inhibitors* tumor necrosis factor inhibitors

^†^calculated chi-square statistic, t-value, z test statistic. No test values are available where Fisher’s exact test was used

^a^smoking and snuff use

^b^education level: low = elementary school, intermediate = high school/vocational education, high = college/university

^c^at least once a month

^d^< 2500 g

^e^preeclampsia/eclampsia/HELLP-syndrome

### Six months postpartum (Table 4)

Six months postpartum 221 women (70%) were breastfeeding. Non-breastfeeding women had higher CRP-scores (5.3 vs 3.3). The women breastfeeding had lower disease activity scores, with a lower mean BASDAI (3.2 vs 4.1) and mean ASDAS-CRP-score (2.1 vs 2.6). The percentage of women reporting inflammatory active disease according to BASDAI-score was also lower for the breastfeeding women (37.6% vs 50.6%). All VAS-scores were significantly lower among the breastfeeding women.

Regarding medication, we found that more women in the non-breastfeeding group used secukinumab or methotrexate at this visit (4 vs 1).

A significantly higher proportion of the women with higher educational level were breastfeeding at six months postpartum (78.2% vs 55.4%). More women in the non-breastfeeding population used tobacco (19%) compared to those breastfeeding (5%). The proportion of women who exercised regularly was higher in the breastfeeding group (66.5% vs 50.6%).

The analysis showed a significant difference in BMI at this visit, with a mean of 25.1 in the breastfeeding group and 27.4 in the non-breastfeeding group. We observed the same when comparing the proportion of overweight and obesity between the groups.

**Table 4:** Differences in breastfeeding and non-breastfeeding mothers with axSpA six months after birth

	**n**	**Breastfeeding** *N* = 221	**n**	**Non-breastfeeding** *N* = 95	*p*-value	**test value**†	**df**
Age, mean (SD)	221	31.9 (4.2)	95	31.6 (3.9)	0.5	0.70	314
Age ≥ 35 years (%)		63 (28.5)		19 (20.0)	0.1	2.50	1
Missing	0						
BMI (kg/m^2^), mean (SD)	204	25.1 (5,0)	89	27.4 (5.1)	< 0.001	−3.75	291
Underweight (< 18.5) (%)		5 (2.5)		0 (0.0)	0.3		
Normal weight (18.5–24.9) (%)		108 (52.9)		33 (37.1)	0.01	6.25	1
Overweight (≥ 25.0) (%)		91 (44.6)		56 (62.9)	0.004	8.31	1
Obesity (≥ 30.0) (%)		28 (13.7)		30 (33.7)	< 0.001	15.58	1
Missing	9						
Tobacco ^a^ (%)	198	9 (4.5)	85	16 (18.8)	< 0.001	15.05	1
Missing	33						
Educational level ^b^	216		92				
Low (%)		2 (0.9)		2 (2.2)	0.59		
Intermediate (%)		45 (20.8)		39 (42.4)	< 0.001	15.11	1
High (%)		169 (78.2)		51 (55.4)	< 0.001	16.44	1
Missing	8						
Working fulltime or part-time (%)	221	15 (6.8)	95	7 (7.4)	0.9	0.04	1
Missing	0						
Exercising regularly ^c^ (%)	170	113 (66.5)	77	39 (50.6)	0.02	5.61	1
Missing	69						
CRP mean (SD)	190	3.3 (4.3)	83	5.3 (8.6)	0.01	−2.51	271
Missing	43						
BASDAI mean (SD)	194	3.2 (2.3)	79	4.1 (2.6)	0.01	−2.731	271
BASDAI ≥ 4 (%)		73 (37.6)		40 (50.6)	0.05	3.91	1
Missing	43						
ASDAS-CRP mean (SD)	145	2.1 (0.9)	64	2.6 (1.1)	< 0.001	−3.43	207
ASDAS-CRP ≥ 1.3 (%)		112 (77.2)		54 (84.4)	0.2	1.38	1
Missing	107						
VAS scores (0–100), mean (SD)							
Pain	188	31.0 (26.3)	83	43.9 (28.9)	< 0.001	−3.31	
Missing	45						
Fatigue	182	37.8 (31.7)	80	46.8 (31.8)	0.03	−2.19	
Missing	54						
Total	188	32.3 (25.7)	82	45.8 (28.6)	< 0.001	−3.54	
Missing	46						
csDMARDs (%)	221	22 (10.0)	95	11 (11.6)	0.7	0.19	1
Sulfasalazine		21 (9.5)		7 (7.4)	0.5	0.38	1
Methotrexate		1 (0.5)		4 (4.2)	0.03		
Missing	0						
TNF-inhibitors (%)	221	118 (53.4)	95	47 (49.5)	0.5	0.41	1
Missing	0						
Secukinumab (%)	221	1 (0.5)	95	4 (4.2)	0.03		
Missing	0						

*n* number of available data on variable, *df* degrees of freedom, *BMI* body mass index, *CRP* C-reactive protein, *BASDAI* bath ankolysing spondylitis disease activity index, *ASDAS* ankylosing spondylitis disease activity score, *VAS* visual analogue scale, *csDMARDs* conventional synthetic disease-modifying anti-rheumatic drugs, *TNF-inhibitors* tumor necrosis factor inhibitors

^†^calculated chi-square statistic, t-value, z test statistic. No test values are available where Fisher’s exact test was used

^a^smoking and snuff use

^b^education level: low = elementary school, intermediate = high school/vocational education, high = college/university

^c^at least once a month

### Twelve months postpartum (Table 5)

Twelve months postpartum 104 women (38%) reported they were breastfeeding. The mean age in the breastfeeding group was slightly higher than the non-breastfeeding group at this visit. The percentage of women aged 35 or older was also significantly higher among the women still breastfeeding (38.5% vs 26.0%). A larger proportion of women with high educational level were breastfeeding (81.0% vs 59.6%). More of the non-breastfeeding women used tobacco compared to the breastfeeding (14% vs 2%).

The only difference in disease variables was the number of women with active disease according to BASDAI-score (30.3% vs 43.4%).

**Table 5:** Differences in breastfeeding and non-breastfeeding mothers with axSpA twelve months after birth

	**n**	**Breastfeeding** ***N*** ** = 104**	**n**	**Non-breastfeeding** ***N*** ** = 169**	*p*-value	**test value**†	**df**
Age mean (SD)	104	33.1 (4.5)	169	32.0 (4.4)	0.05	1.97	271
Age ≥ 35 years (%)		40 (38.5)		44 (26.0)	0.03	4.67	1
Missing	0						
BMI, mean (SD)	96	25.0 (4.9)	157	25.7 (5.6)	0.3	−1.03	251
Underweight (< 18.5) (%)		3 (3.1)		4 (2.5)	1.0		
Normal weight (18.5–24.9) (%)		56 (58.3)		85 (54.1)	0.5	0.43	1
Overweight (≥ 25.0) (%)		37 (38.5)		68 (43.3)	0.5	0.56	1
Obesity (≥ 30.0) (%)		12 (12.5)		32 (20.4)	0.1	2.58	1
Missing	20						
Tobacco ^a^ (%)	96	2 (2.1)	156	22 (14.1)	0.002	9.97	1
Missing	21						
Educational level ^b^	100		166				
Low (%)		3 (3.0)		3 (1.8)	0.68		
Intermediate (%)		16 (16.0)		64 (38.6)	< 0.001	15.10	1
High (%)		81 (81.0)		99 (59.6)	< 0.001	13.02	1
Missing	7						
Working fulltime or part-time (%)	104	59 (56.7)	169	89 (52.7)	0.5	0.43	1
Missing	0						
Exercising regularly ^c^ (%)	88	58 (65.9)	139	95 (68.3)	0.7	0.15	1
Missing	46						
CRP mg/L mean (SD)	88	4.7 (9.0)	146	4.2 (5.9)	0.5	0.60	232
Missing	39						
BASDAI mean (SD)	89	3.0 (2.3)	143	3.6 (2.6)	0.08	−1.78	230
BASDAI ≥ 4 (%)		27 (30.3)		62 (43.4)	0.05	3.93	1
Missing	41						
ASDAS-CRP mean (SD)	65	2.1 (0.9)	111	2.4 (1.0)	0.06	−1.92	174 b
ASDAS-CRP ≥ 1.3 (%)		50 (78.1)		92 (82.9)	0.4	0.60	1
Missing	98						
VAS scores (0–100) mean (SD)							
Pain	90	29.2 (23.4)	145	35.9 (28.8)	0.2	−1.37	
Missing	38						
Fatigue	89	38.0 (31.3)	142	45.4 (33.5)	0.1	−1.66	
Missing	42						
Total	86	36.3 (25.9)	142	40.2 (29.5)	0.4	−0.80	
Missing	45						
csDMARDs (%)	104	8 (7.7)	169	17 (10.1)	0.5	0.43	1
Sulfasalazine		8 (7.7)		13 (7.7)	1.0	0.00	1
Methotrexate		0 (0.0)		5 (3.0)	0.2		
Missing	0						
TNF-inhibitors (%)	104	64 (61.5)	169	89 (52.7)	0.2	2.06	1
Missing	0						
Secukinumab (%)	104	1 (1.0)	169	4 (2.4)	0.7		
Missing	0						

*n* number of available data on variable, *df* degrees of freedom, *BMI* body mass index, *CRP* C-reactive protein, *BASDAI* bath ankolysing spondylitis disease activity index, *ASDAS* ankylosing spondylitis disease activity score, *VAS* visual analogue scale, *csDMARDs* conventional synthetic disease-modifying anti-rheumatic drugs, *TNF-inhibitors* tumor necrosis factor inhibitors

^†^calculated chi-square statistic, t-value, z test statistic. No test values are available where Fisher’s exact test was used

^a^smoking and snuff use

^b^education level: low = elementary school, intermediate = high school/vocational education, high = college/university

^c^at least once a month

## Discussion

The current study showed that a large proportion of women with axSpA were breastfeeding at six weeks and six months after giving birth. The proportions were respectively 86% and 70%. Factors negatively associated with breastfeeding were inflammatory active disease and poor scores of self-reported health status. A larger proportion of the non-breastfeeding women had given birth with C-section. We found no differences between the groups regarding working status or the use of TNF-inhibitors and sulfasalazine.

A previous study about breastfeeding in the general Norwegian population, conducted by the Norwegian Directorate of Health and Statistics Norway (SSB), showed that 95% of the mothers were breastfeeding two weeks after birth and 81% were still breastfeeding after four months [14]. Further, after seven months 67% were still breastfeeding, and 35% after twelve months [14]. This is similar to the percentages found in our study, though we have not compared the proportions statistically.

The proportion of women breastfeeding in our cohort could be influenced by the lengthy maternal leave in Norway and may not be generalizable to the same patients in other countries. Maternity wards and Child and Maternal health centers in Norway may also have an impact on these numbers, by strongly encouraging women to breastfeed and having prioritized information on lactation for many years [15]. In Norway, parents are entitled to a total of 12 months of paid parental leave postpartum, funded by the Norwegian government. This period includes the exclusive maternal leave up to 12 weeks during pregnancy and six weeks after birth [16]. Previous studies have shown that breastfeeding rates are influenced by the length of parental leave, both when comparing countries [17] and states in the USA with different legislation [18]. In the United Kingdom the statutory maternity leave is 52 weeks made up of 26 weeks ordinary maternity leave and 26 weeks additional maternity leave [19]. Statutory maternity leave in the United Kingdom is paid by the government up to 39 weeks. In contrast, in the United States the Family and Medical Leave Act (FMLA) only provides certain employees with up to 12 weeks of unpaid, job-protected leave per year [20], although many states have expanded or added to this federal legislation with their own family leave policies [21].

### Delivery method

A higher proportion of the non-breastfeeding group delivered by C-section compared to the breastfeeding women. A previous study on systemic lupus erythematosus (SLE) and breastfeeding also reported that C-section was associated with not initiating lactation [22]. Barriers to initiate breastfeeding after C-section may be physical limitations such as surgical incision pain [23]. The operative birth-method of C-section could also cause stress for both mother and child and therefore be a reason for not initiating breastfeeding. C-section procedure often requires uterotonics and analgesics in high doses, which may also have a negative impact on the initiation of breastfeeding [24]. A Norwegian study on pregnancy outcomes in patients with axSpA published in 2023 observed an increased risk of elective C-section in women with axSpA, and that elective C-section was more frequent in inflammatory active axSpA [25].

### Disease activity and self-reported data on health status

Non-breastfeeding women had higher disease activity measured by ASDAS-CRP than the breastfeeding women both six weeks and six months postpartum. Additionally, at the six month follow up, the non-breastfeeding women had higher disease activity measured by BASDAI score and a higher mean CRP. Both at six weeks and six months postpartum the non-breastfeeding group also reported more pain according to VAS, and at six months the same group also reported more fatigue. The higher disease activity may be a reason why fewer of these women were breastfeeding. Lower disease activity among the breastfeeding could also indicate that breastfeeding contributes to reduced inflammation, as a study suggested for rheumatoid arthritis [26].

### Medication

The use of both methotrexate and secukinumab was more frequent among the non-breastfeeding women six months postpartum (Table 6). Methotrexate has not been recommended during breastfeeding [5–7]. Secukinumab was not considered compatible with breastfeeding until recently [27]. A large proportion of our population gave birth while it was still not recommended to use secukinumab during lactation. This could explain why the use of this medication was more common among the non-breastfeeding women.

**Table 6:** Use of medication at inclusion, six weeks, six and twelve months postpartum

	**Inclusion** ***N* = 417**	**6 weeks postpartum** ***N* = 417**	**6 months postpartum** ***N* = 324**	**12 months postpartum *N* = 290**
csDMARSs (%)	27 (6.5%)	27 (6.5%)	33 (10.2%)	26 (9.0%)
Sulfasalazine	22 (5.3%)	27 (6.5%)	28 (8.6%)	22 (7.6%)
Methotrexate	5 (1.2%)	1 (0.2%)	5 (1.5%)	5 (1.7%)
TNF-inhibitors (%)	116 (27.8%)	125 (30.0%)	166 (51.2%)	163 (56.2%)
Secukinumab (%)	0 (0.0%)	4 (1.0%)	5 (1.5%)	5 (1.7%)

*csDMARDs* conventional synthetic disease-modifying anti-rheumatic drugs, *TNF-inhibitors* tumor necrosis factor inhibitors

The use of TNF-inhibitors did not differ between the breastfeeding and the non-breastfeeding group at any of the follow ups, indicating that the patients were well informed about medications compatible with breastfeeding. We observed that women with inflammatory active disease according to both ASDAS-CRP and BASDAI were less likely to use TNF-inhibitors at the six and twelve months follow up. This could indicate that some women did not receive optimal medical treatment.

### Demographic data

Breastfeeding women had higher mean age both six weeks and twelve months postpartum compared to non-breastfeeding women. Previous research has demonstrated an association between increased maternal age and breastfeeding rates in a population-based study from Norway [14, 28].

During all registration fewer breastfeeding women used tobacco than the non-breastfeeding group. Neither smoking nor snuff use is recommended during breastfeeding [29].

Working status was the only variable that did not show any significant differences between the breastfeeding and non-breastfeeding groups at any of the visits. This is an interesting finding since it shows that breastfeeding in this population was not affected by work. Norwegian women have a right to get paid time off during their workday to breastfeed [16]. This might be a reason why there was no difference in this variable.

Mean BMI was higher for the non-breastfeeding women at six months. This association is well-established, as earlier studies have indicated a negative correlation between maternal obesity and duration of breastfeeding [30].

### Strengths

Being one of very few studies looking at breastfeeding in women with axSpA, the current study may contribute new information on the subject. One strength of our study was the high number of patients included. We had access to a large number of variables and collected data for a long period of time, making our patient population and amount of data comprehensive. Furthermore, all the patients included had a similar demographic background and were included in the register in the same way.

### Limitations

The RevNatus register does not collect detailed information regarding breastfeeding. As a result, we did not have data explaining why a patient did or did not breastfeed. There may be additional factors influencing this choice that we are unaware of. Further, our data did not specify whether the women breastfed exclusively or partially. According to national Norwegian guidelines, exclusive breastfeeding is recommended the first 6 months for mothers able to do so [14]. Later a combination of breastfeeding and other nutrition is common [14].

A limitation was the lack of a healthy population group to compare the axSpA patients to. Our population was very homogenous, and new studies from other countries may find other results.

Another limitation was missing data, especially for the variable ASDAS-CRP. Differences between the groups in the use of methotrexate and secukinumab should be interpreted with caution, as the results are based on a small amount of data. All the patients included in the study attended the six weeks postpartum follow up, but some did not have data available from the six month and twelve month follow up. This made it difficult to compare findings from the different follow-ups. The collection of data from different follow-ups was nevertheless an advantage. This made it possible for us to compare the population at different timepoints. Missing data regarding the recruitment of our patient population could be a limitation. By using data from RevNatus, we know the number of patients initially recruited. The number of patients that were approached for inclusion and declined is unknown. Some women eligible for the register may never have been approached, not having been referred to a rheumatological department eg because of inactive disease. This could have led to a skewed distribution in our cohort regarding disease activity, if more women with high disease activity were approached.

The measures of disease activity and self-reported health status used in our study have certain limitations. ASDAS-CRP and BASDAI are both recommended measures of disease activity in axSpA [12]. BASDAI has been in use longer than ASDAS-CRP in both clinical follow-up and research, but is based exclusively on self-reported data from the patients. ASDAS-CRP is considered more objective, combining self-reported items with CRP [12]. For some patients, the CRP blood test was not conducted during follow ups, which means that ASDAS-CRP cannot be assessed. The VAS variables are subjective and not disease-specific variables.

CRP, used both alone and in relation to ASDAS-CRP in this study, is a more objective measure of disease activity than the self-reported items. Unlike erythrocyte sedimentation rate (ESR), CRP is not influenced by pregnancy or breastfeeding [31]. It may however be influenced by other causes of inflammation and infection in the body, such as mastitis.

In our cohort 70% of the population had a high educational level, which could indicate a selection bias, that women with higher levels of education were more willing to join the register. All patients eligible for RevNatus are offered to join the register, regardless of their education level. The percentage of highly educated women in Norway is high [32]**,** though not as high as in our cohort, suggesting a skewed distribution regarding socio-economic status in our cohort. As mentioned in our results, a significant association between high levels of education and breastfeeding was found for all follow-ups. Several other studies have found the same association between educational level and lactation [33].

In this prospective study we compared breastfeeding and non-breastfeeding women at three different points in time. We did not follow the cases longitudinally and have not censored for reasons of loss to follow up, such as stopping breastfeeding, not attending visits or new pregnancies and have therefore not used multiple testing. No adjustments were made for multiple comparisons.

Further research on the subject should look more deeply into specific reasons why women with axSpA do not breastfeed, as well as how the proportion of breastfeeding patients may become higher.

## Conclusion

Our study demonstrated that a large proportion of women with axSpA were breastfeeding at six weeks and six months after giving birth. Disease activity scores, as well as self-reported data on health status, were worse for women not breastfeeding during all visits postpartum. A larger proportion of the non-breastfeeding group had given birth with C-section.

Women with axSpA should be encouraged to breastfeed, and improved disease management might enhance the likelihood of these women breastfeeding. Patients who have delivered with C-section and those with inflammatory active disease should receive additional guidance regarding breastfeeding.

## Data Availability

Data that support the findings of this study are obtained from the nationwide quality register RevNatus and were used under the licence for the current study. Due to the requirements of the involved register and the general data protection regulations, the data cannot be shared publicly. However, they may be obtained from the authors upon reasonable request and with the permission of RevNatus.

## Abbreviations

ASDAS-CRP: Ankylosing Spondyloarthritis Disease Activity Index-CRP
axSpA: Axial Spondyloarthritis
BASDAI: Bath Ankylosing Disease Activity Index
bDMARDs: Biologic DMARDs
BMI: Body Mass Index
csDMARDs: Conventional synthetic DMARDs
CRP: C-reactive protein
C-section: Caesarean section
df: Degrees of freedom
DMARDs: Disease Modifying Anti-Rheumatic Drugs
ESR: Erythrocyte sedimentation rate
HELLP: Hemolysis, Elevated Liver enzymes and Low Platelets
MTX: Methotrexate
nr-axSpA: Non-radiographic axSpA
NSAIDs: Non-Steroidal Anti-Inflammatory Drugs
r-axSpA: Radiographic axSpA
SLE: Systemic lupus erythematosus
SpA: Spondyloarthritis
SSB: Statistics Norway
TNFi: Tumor Necrosis Factor-inhibitors
VAS: Visual analogue scales
